# Evaluation of Cesarean Rates for Term, Singleton, Live Vertex Deliveries in China in 2020 Among Women With No Prior Cesarean Delivery

**DOI:** 10.1001/jamanetworkopen.2023.4521

**Published:** 2023-03-23

**Authors:** Shaohua Yin, Lian Chen, Yubo Zhou, Pengbo Yuan, Xiaoyue Guo, Jie Lu, Lin Ge, Huifeng Shi, Xiaoxia Wang, Luyao Li, Jie Qiao, Yangyu Zhao, Hongbo Qi, Xudong Ma, Yuan Wei

**Affiliations:** 1 Department of Obstetrics and Gynecology, Peking University Third Hospital, Beijing, China; 2 National Clinical Research Centre for Obstetrics and Gynaecology, Beijing, China; 3 National Centre for Healthcare Quality Management in Obstetrics, Beijing, China; 4 Institute of Reproductive and Child Health, National Health Commission Key Laboratory of Reproductive Health, Peking University Health Science Centre, Beijing, China; 5 Department of Obstetrics, Women and Children’s Hospital of Chongqing Medical University, Chongqing, China; 6 Department of Healthcare Quality Evaluation, Bureau of Medical Administration, National Health Commission of the People’s Republic of China, Beijing, China

## Abstract

**Importance:**

Substantial regional variation in cesarean delivery rates has been reported in China, but there is a lack of reports on hospital-level variation in these rates among low-risk deliveries.

**Objectives:**

To evaluate hospital variation in cesarean rates in China for term, singleton, live vertex deliveries among women with no prior cesarean delivery and to estimate contributions of individual and hospital factors.

**Design, Setting, and Participants:**

This nationwide cross-sectional study used data from maternal patient discharge records collected by the Hospital Quality Monitoring System in China from January 1 to December 31, 2020. Pregnant female individuals aged 15 to 49 years (referred to hereafter as women) with at least 1 live birth were included, and low-risk deliveries were defined as term, singleton, live, vertex deliveries with no prior cesarean delivery.

**Exposures:**

Birth by cesarean delivery.

**Main Outcomes and Measures:**

The main outcome was cesarean delivery rate by hospital. Hierarchical logistic regression analysis was used to calculate the adjusted cesarean rate and to estimate the percentage of hospital variation in low-risk deliveries explained by individual and hospital factors.

**Results:**

Among the 7 635 149 deliveries identified from 4359 hospitals in 31 provinces of mainland China, 6 599 468 (86.4%) were considered low risk. Of overall and low-risk deliveries, 3 400 162 and 2 638 097 were cesarean deliveries, corresponding to mean rates of 44.5% and 40.0%, respectively. The mean (SD) maternal age for overall and low-risk deliveries was 29.1 (4.0) and 28.8 (4.8) years, respectively, and mothers were more likely to be of Han ethnicity (89.5%). Cesarean rates varied widely among hospitals, with absolute differences between the 5th and 95th percentiles of 53.5% (19.4%-72.9%) for overall deliveries and 56.8% (14.3%-71.1%) for low-risk deliveries. Large absolute differences remained after adjusting for maternal characteristics, with rates of 47.4% (19.1%-66.5%) for overall deliveries and 52.6% (15.0%-67.6%) for low-risk deliveries. Among low-risk deliveries, hospital factors (eg, hospital province location) explained 31.3% of the hospital variation in cesarean rate and individual factors explained an additional 2.0%.

**Conclusions and Relevance:**

The findings of this cross-sectional study suggest that cesarean rates varied markedly among hospitals in China in 2020, which may be attributable to hospital rather than individual factors. Future work is needed to design hospital-level initiatives to optimize cesarean use, particularly among low-risk deliveries.

## Key Points

**Question:**

Do cesarean delivery rates vary among hospitals in China?

**Findings:**

In this cross-sectional study of more than 7.6 million deliveries from 4359 hospitals in China in 2020, the 5th and 95th percentiles of cesarean rates varied from 19% to 67% for overall deliveries and from 15% to 68% for term, singleton, live, vertex deliveries among women with no prior cesarean delivery. This large variation was likely driven by hospital factors, such as province location, rather than individual factors.

**Meaning:**

The results of this study suggest that cesarean delivery rates varied substantially among hospitals in China in 2020, reflecting inconsistent cesarean use and potential cesarean overuse even in low-risk deliveries.

## Teaser

This cross-sectional study evaluates hospital variation in cesarean rates in China in 2020 for term, singleton, live, vertex deliveries among women with no prior cesarean delivery.

## Introduction

Reducing the rate of cesarean delivery has been a public health focus in many nations, as cesarean overuse can be harmful to both mothers and their offspring^1^ and, in particular, can complicate future pregnancies.^2^ With the largest population and the second largest annual number of births worldwide,^3^ the cesarean delivery rate in China has drawn global concern since the World Health Organization reported a rate of up to 46.2% during 2007 to 2008.^4^ Between 2012 and 2016, the rate declined slightly, possibly due to a variety of initiatives taken by the Chinese government to curb cesarean use.^5^ Thereafter, the rate rebounded, which is likely attributable to the end of some initiatives and a growing proportion of older multiparous women with a prior cesarean delivery following implementation of the 2-child policy in China in 2016.^6^ Since 2018, the cesarean delivery rate has been surpassing 40% in urban areas of China and has even approached 35% in rural areas,^6^ far exceeding the benchmarks of 15% or 19%.^7,8^ In 2021, a 3-child policy was implemented in China,^9^ which may lead to further changes in maternal characteristics. Given the adverse health effects of cesarean delivery, control of its overuse must be reiterated.

Hospital-level variations in cesarean rates have important implications for local and national health policies that would likely target hospitals with high cesarean rates.^10,11,12^ However, little attention has been paid to hospital variation in cesarean rates in China, where 99.7% of deliveries occurred in hospitals as early as 2015.^13^ The cesarean rate among women with low delivery risk (ie, those with term gestation, with a live singleton baby in vertex presentation, and with no prior cesarean delivery^14^) has been widely adopted as an important obstetric quality measure of a hospital.^15^ Variation in this measure among hospitals has been investigated extensively in high-income countries^15,16,17,18^ but is lacking in low- and middle-income countries, including China. In addition, the extent to which individual and hospital characteristics contribute to this variation remains poorly understood, as previous studies have yielded inconsistent results and have been limited to high-income countries.^17,19,20^ Improved understanding of hospital variation in cesarean delivery rates in China and identification of sources of this variation will guide a safe reduction in the use of this procedure and aid in future quality improvement efforts.

Using individual data from 4359 hospitals in 31 provinces of mainland China, we evaluated between-hospital variation in cesarean rates among both overall and low-risk deliveries. In addition, we estimated the contributions of individual and hospital factors to hospital variation in rates of low-risk deliveries.

## Methods

This study was approved by the Peking University Third Hospital Medical Science Research Ethics Committee. Informed consent was waived because deidentified data were used. The study followed the Strengthening the Reporting of Observational Studies in Epidemiology (STROBE) reporting guideline.

### Data Source

This cross-sectional study used individual patient discharge data from the Hospital Quality Monitoring System (HQMS), a national data collection system established by the National Health Commission of China in 2011 to improve the quality of clinical services. The HQMS database contains both administrative data and clinical information, making it one of the most comprehensive national sources of information on hospital-based care in China; it has been used regularly in health services research.^21,22,23^ The database originally covered most referral hospitals nationwide (those providing medical services to the whole country beyond cities and provinces, with the capability to assist patients with complicated conditions referred from lower levels); in 2020, it began to cover nonreferral hospitals (those providing medical services to multiple communities within a region, primarily serving patients with noncomplicated conditions). Covered hospitals are required to submit daily individual-level data on the front page of inpatient discharge records to the HQMS database, which contains approximately 346 variables, including information on maternal sociodemographic characteristics, admission and discharge diagnoses, and hospital characteristics.^21,22,23^ The HQMS database includes quality control procedures to ensure the integrity, logicality, and accuracy of data entry. Once an error is identified, the data are returned and the hospital is informed to check and correct the data.^24^

### Data Extraction and Cleaning

Maternal characteristics (including maternal age, ethnicity, marital status [married, single, or divorced], medical insurance status, history of cesarean delivery, number of fetuses, fetal presentation, pregnancy complications, perinatal outcomes, and delivery mode) and hospital characteristics (including hospital level, hospital specialization, and hospital province location) were extracted from the HQMS database. Ethnicity was self-reported as Han or Chinese ethnic minority (eg, Zhuang, Hui, Manchu, Uighur, or other ethnicity); these data were collected owing to a previously reported association between ethnicity and cesarean rates.^25^ In all, records for 8 042 773 pregnant women with at least 1 live birth (between January 1 and December 31, 2020) were extracted initially. Pregnant women were excluded if they were not Chinese or were of an unidentified nationality (n = 38 133), were aged younger than 15 or older than 49 years (referred to hereafter as women; n = 149 682), or had missing data on ethnicity, marital status, or medical insurance status (n = 201 429). To ensure sufficient volume per hospital for comparison, pregnant women from a hospital with fewer than 100 deliveries (n = 18 380) were also excluded. The analysis included 7 635 149 pregnant women (94.9%) from 2734 of 5243 nonreferral hospitals and 1625 of 2838 referral hospitals (eFigure 1 in Supplement 1), representing more than 50.0% of nonreferral and referral hospitals in China in 2020.^26^

### Definition of Variables

Cesarean delivery was identified using *International Classification of Diseases, Ninth Revision, Clinical Modification* codes (74.0, 74.1, 74.2, 74.4, and 74.9) and *International Statistical Classification of Diseases, Tenth Revision* codes (O60, O82, O84, O86, O90, and P03), as detailed in eTable 1 in Supplement 1. Cesarean rate was calculated as the number of cesarean deliveries divided by the number of total deliveries. Rates were calculated for pregnant women overall and for pregnant women with low delivery risk defined as those with term, singleton, live birth deliveries in vertex presentation and with no prior cesarean delivery, according to the definition developed by the Agency for Healthcare Research and Quality (AHRQ).^14^

Hospitals were classified into 4 geographic regions according to their province location (northeastern, eastern, central, and western regions), according to the method of the National Bureau of Statistics of China.^27^

### Statistical Analysis

Results are expressed as means (SDs) or counts (percentages). Comparisons between groups were made using the *t* test for continuous variables and the χ^2^ test for categorical variables. Cesarean rates among overall and low-risk deliveries were first calculated for each hospital. Descriptive analyses of the 5th, 25th, 50th (medians), 75th, and 95th percentiles of cesarean rates were performed to assess between-hospital variation. To control for individual factors that may have contributed to hospital variation, the adjusted cesarean rate of each hospital was calculated using hierarchical logistic regression models.^28^ Adjusted individual factors included maternal age (<35 or ≥35 years), ethnicity (Han or Chinese ethnic minority), marital status (married, single, or divorced), medical insurance status (yes or no), and pregnancy complications (yes or no). Funnel plots were drawn to examine whether hospital variation in cesarean rates exceeded that expected from random fluctuations alone.^29^ Box-and-whisker plots were generated to visualize hospital variation among hospital subgroups defined by hospital level (nonreferral or referral), hospital specialization (maternity specialized or general), hospital delivery volume (100-999, 1000-2999, 3000-4999, or ≥5000), and geographic region (northeastern, eastern, central, or western).

To explore individual and hospital factors that may explain hospital variation in cesarean delivery rate, hierarchical logistic regression models were fitted to individual-level data for low-risk deliveries. The first unadjusted model included only hospital-specific random intercepts, which estimated the baseline variability in cesarean rates among hospitals after subtracting the effects of random sampling variation (between-hospital variance). Then this model was extended to adjust for individual and hospital factors. The variation attributed to individual or hospital factors was estimated by calculating the reduction, if any, in between-hospital variance from unadjusted to adjusted models. The fully adjusted model included individual and hospital factors that were associated with the risk of cesarean delivery. Individual factors included maternal age, ethnicity, marital status, medical insurance status, and pregnancy complications; hospital factors included hospital level, hospital specialization, hospital delivery volume, and hospital province location. The Hosmer-Lemeshow test was used to assess the goodness of fit of the adjusted model. To facilitate interpretation, estimated odds ratios and their corresponding 95% CIs were converted to relative risks (RRs) (eTable 2 in Supplement 1).^30^

Data analyses were performed using SAS, version 9.4 (SAS Institute Inc). A 2-sided *P* < .05 was considered statistically significant. Statistical analysis was performed on March 17, 2022.

## Results

In this study, a total of 7 635 149 deliveries (accounting for 63.0% of all deliveries in China) were reported to the HQMS between January 1 and December 31, 2020, from 4359 hospitals in 31 provinces of China. Of the 7 635 149 pregnant women included, 6 599 468 (86.4%) were identified as being at low risk for cesarean delivery. Among overall and low-risk deliveries, the mean (SD) maternal age was 29.1 (4.0) and 28.8 (4.8) years, respectively; mothers were also more likely to be of Han ethnicity (89.5% compared with 10.5% Chinese ethnic minority for both delivery types), to be married (96.3% for both delivery types), and to have a medical insurance (67.6% and 67.9%; Table 1).

**Table 1.** Individual and Hospital Characteristics Among Overall Deliveries and Low-risk Deliveries at 4359 Hospitals in China in 2020^a^

Characteristic	Overall		Low risk	
	Delivery (n = 7 635 149)^b^	Cesarean delivery (n = 3 400 162)^c^	Delivery (n = 6 599 468)^b^	Cesarean delivery (n = 2 638 097)^c^
Maternal age, y, mean (SD)	29.1 (4.0)	30.1 (4.9)	28.8 (4.8)	29.8 (4.8)
<35	6 620 697 (86.7)	2 792 457 (42.2)	5 862 968 (88.8)	2 228 533 (38.0)
≥35	1 014 452 (13.3)	607 705 (59.9)	736 500 (11.2)	409 564 (55.6)
Ethnicity				
Han	6 836 387 (89.5)	3 089 454 (45.2)	5 908 513 (89.5)	2 400 715 (40.6)
Chinese ethnic minority^d^	798 762 (10.5)	310 708 (38.9)	690 955 (10.5)	237 382 (34.4)
Marital status				
Married	7 352 525 (96.3)	3 303 464 (44.9)	6 352 575 (96.3)	2 563 543 (40.4)
Single	259 421 (3.4)	84 688 (32.6)	228 508 (3.5)	66 015 (28.9)
Divorced	23 203 (0.3)	12 010 (51.8)	18 385 (0.3)	8539 (46.4)
Medical insurance status				
Yes	5 161 215 (67.6)	2 343 398 (45.4)	4 477 926 (67.9)	1 829 859 (40.9)
No	2 473 934 (32.4)	1 056 764 (42.7)	2 121 542 (32.1)	808 238 (38.1)
Gestational age				
Term	7 270 398 (95.2)	3 183 868 (43.8)	6 599 468 (100)	2 638 097 (40.0)
Preterm	364 751 (4.8)	216 294 (59.3)	0	NA
No. of fetuses				
Singleton	7 394 269 (96.8)	3 227 771 (43.7)	6 599 468 (100)	2 638 097 (40.0)
Multiple pregnancy	240 880 (3.2)	172 391 (71.6)	0	NA
Prior cesarean delivery				
No	7 437 759 (97.4)	3 234 373 (43.5)	6 599 468 (100)	2 638 097 (40.0)
Yes	197 390 (2.6)	165 789 (84.0)	0	NA
Fetal presentation				
Cephalic	7 271 777 (95.2)	3 079 383 (42.3)	6 599 468 (100)	2 638 097 (40.0)
Breech or other	363 372 (4.8)	320 779 (88.3)	0	NA
Pregnancy complications^e^				
No	5 942 236 (77.8)	2 316 976 (39.0)	5 249 737 (79.5)	1 843 683 (35.1)
Yes	1 692 913 (22.2)	1 083 186 (64.0)	1 349 731 (20.5)	794 414 (58.9)
Hospital delivery volume				
100-999	962 807 (12.6)	448 764 (46.6)	1 039 090 (15.8)	445 640 (42.9)
1000-2999	4 709 881 (61.7)	2 111 142 (44.8)	4 182 039 (63.4)	1 693 383 (40.4)
3000-4999	1 125 419 (14.7)	462 446 (41.1)	924 522 (14.0)	331 840 (35.9)
≥5000	837 042 (11.0)	377 810 (45.1)	453 817 (6.9)	167 234 (36.9)
Hospital level				
Referral	4 304 446 (56.4)	2 026 863 (47.1)	3 595 292 (54.5)	1 489 928 (41.4)
Nonreferral	3 330 703 (43.6)	1 373 299 (41.2)	3 004 176 (45.5)	1 148 169 (38.2)
Hospital specialization				
General	5 519 261 (72.3)	2481743 (45.0)	4 816 068 (73.0)	1 966 555 (40.8)
Maternity specialized	2 115 888 (27.7)	918 419 (43.4)	1 783 400 (27.0)	671 542 (37.7)
Geographic region^f^				
Northeastern	260 768 (3.4)	150 281 (57.6)	225 000 (3.4)	121 296 (53.9)
Eastern	3 265 584 (42.8)	1 389 534 (42.6)	2 778 529 (42.1)	1 039 012 (37.4)
Central	1 765 583 (23.1)	858 636 (48.6)	1 570 951 (23.8)	706 684 (45.0)
Western	2 343 214 (30.7)	1 001 711 (42.8)	2 024 988 (30.7)	771 105 (38.1)

Abbreviation: NA, not applicable (deliveries were excluded for this analysis).

^a^ Term, singleton, live, vertex deliveries with no prior cesarean delivery.

^b^ Data are presented as No. (%) of deliveries unless otherwise indicated.

^c^ Data are presented as No. (rate) of cesarean deliveries unless otherwise indicated. The rate of cesarean delivery between maternal and hospital characteristics was examined using χ^2^ tests.

^d^ Includes Zhuang, Hui, Manchu, Uighur, and other ethnicity.

^e^ Pregnancy complications included gestational hypertension, preeclampsia, placental accreta, placenta previa, placental abruption, antenatal hemorrhage, birth canal morphologic deformity, cord prolapse, macrosomia, or other.

^f^ Four geographic regions were classified based on hospital province location as follows: northeastern (Heilongjiang, Jilin, and Liaoning), eastern (Beijing, Fujian, Guangdong, Hainan, Hebei, Jiangsu, Shanghai, Tianjin, and Zhejiang), central (Anhui, Henan, Hubei, Hunan, Jiangxi, and Shanxi), and western (Chongqing, Gansu, Guangxi, Guizhou, Inner Mongolia, Ningxia, Qinghai, Shaanxi, Sichuan, Tibet, Xinjiang, and Yunnan).

**Figure 1.**
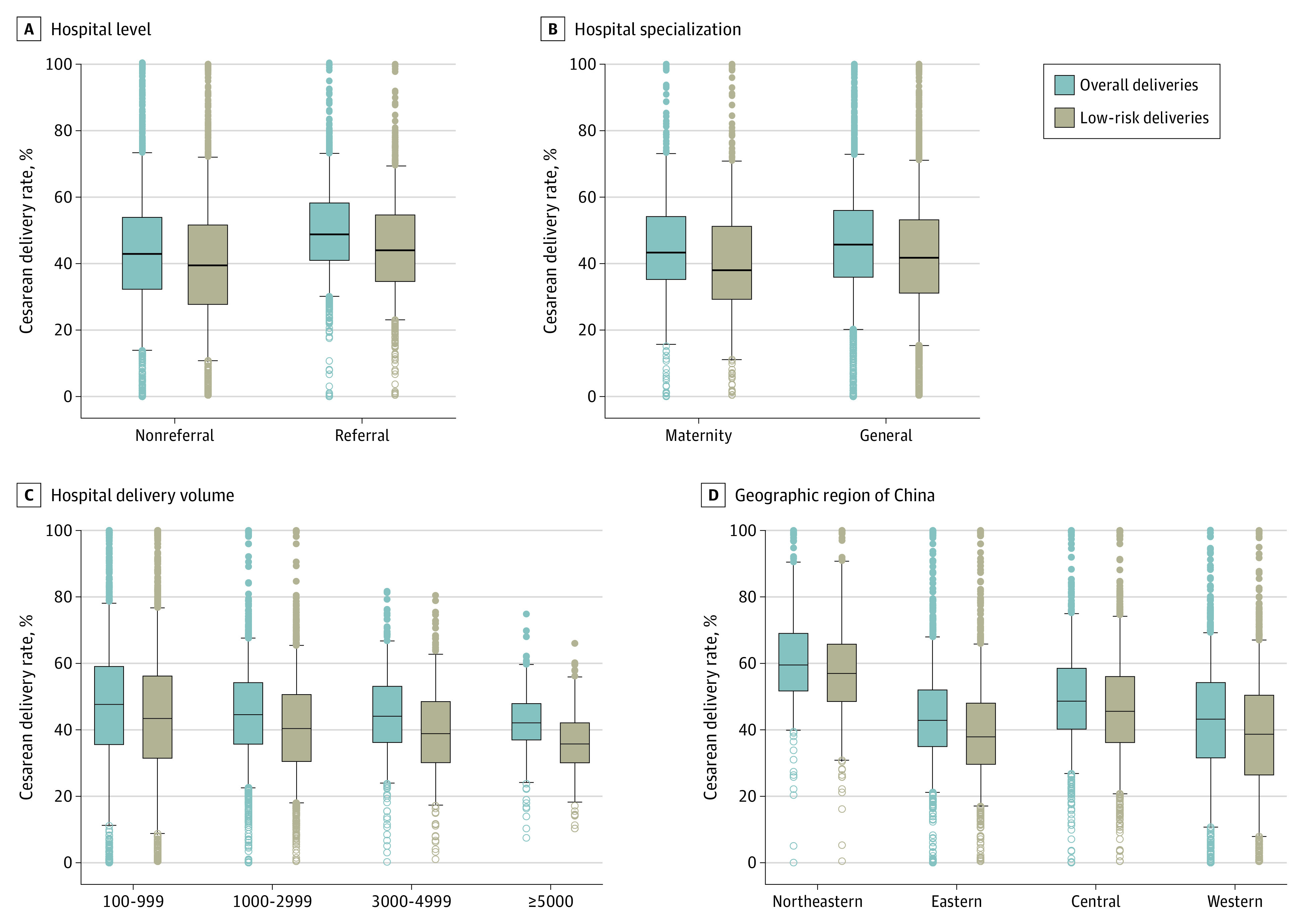
Overall and Low-risk Cesarean Delivery Rates by Hospital Level (A), Hospital Specialization (B), Hospital Delivery Volume (C), and Geographic Region (D) in China in 2020 For each box-and-whisker plot, the horizontal bar indicates the median, the upper and lower limits of the boxes indicate the IQR, and the ends of the whiskers from the bottom of the box to the top indicate the 5th and 95th percentiles. Shaded boxes represent the rates for overall and low-risk deliveries. Open and filled circles represent rates less than the 5th percentile or greater than the 95th percentile, respectively. All *P* values for comparison of cesarean rates among hospital characteristics were <.001, according to Kruskal-Wallis tests.

Among overall and low-risk deliveries during this period, there were 3 400 162 and 2 638 097 cesarean deliveries, corresponding to mean cesarean rates of 44.5% and 40.0%, respectively. Cesarean rates differed according to individual and hospital characteristics (Table 1), and they varied substantially among the 4359 hospitals, far exceeding those expected by random fluctuation (eFigure 2 in Supplement 1). Absolute differences between the 5th and 95th percentiles of cesarean rates were 53.5% (19.4%-72.9%) for overall deliveries and 56.8% (14.3%-71.1%) for low-risk deliveries (Table 2). After adjustment for individual factors, hospital variations in cesarean rates decreased but were still pronounced (Table 2). Marked hospital variations persisted in subgroups stratified by hospital characteristics (Figure 1). Variations narrowed but remained large as hospital delivery volume increased. For example, absolute differences between overall and low-risk cesarean rates were 45.1% (22.5%-67.6%) and 47.8% (17.6%-65.4%) in hospitals with 1000 to 2999 deliveries and 41.0% (24.0%-65.0%) and 43.8% (17.3%-61.1%) in hospitals with 3000 or more deliveries (Figure 1). Absolute differences were larger for nonreferral hospitals (59.0% for overall deliveries vs 61.6% for low-risk deliveries) than for referral hospitals (42.7% vs 46.7%) and for maternity-specialized hospitals (57.4% vs 60.2%) than for general hospitals (52.7% vs 56.2%); the variations persisted in subgroups jointly stratified by hospital specialization and hospital level (eTable 3 in Supplement 1). In subgroup analysis by geographic region, the absolute difference was largest in hospitals in the western region (58.4% for overall deliveries vs 59.5% for low-risk deliveries), followed by hospitals in the northeastern (50.6% vs 60.3%) and central (48.1% vs 53.9%, respectively) regions, and it was lowest in the eastern region (46.8% vs 49.1%). Notably, hospital variation in low-risk cesarean rates was larger than that in overall cesarean rates (Table 2), which persisted in subgroups stratified by hospital characteristics (Figure 1 and eTable 3 in Supplement 1).

**Table 2.** Distribution of Overall and Low-risk Cesarean Delivery Rates Among 4359 Hospitals in China in 2020

Cesarean rate	Percentile, %					Difference, %^a^
	5th	25th	50th	75th	95th	
Unadjusted						
Overall deliveries	19.4	35.8	45.3	55.8	72.9	53.5
Low-risk deliveries	14.3	30.7	41.2	52.8	71.1	56.8
Adjusted^b^						
Overall deliveries	19.1	32.7	38.8	44.9	66.5	47.4
Low-risk deliveries	15.0	30.6	37.8	44.3	67.6	52.6

^a^ Calculated by subtracting the 5th percentile from the 95th percentile.

^b^ Adjusted for maternal sociodemographic and clinical characteristics, including maternal age, ethnicity, marital status, medical insurance status, and pregnancy complications.

**Figure 2.**
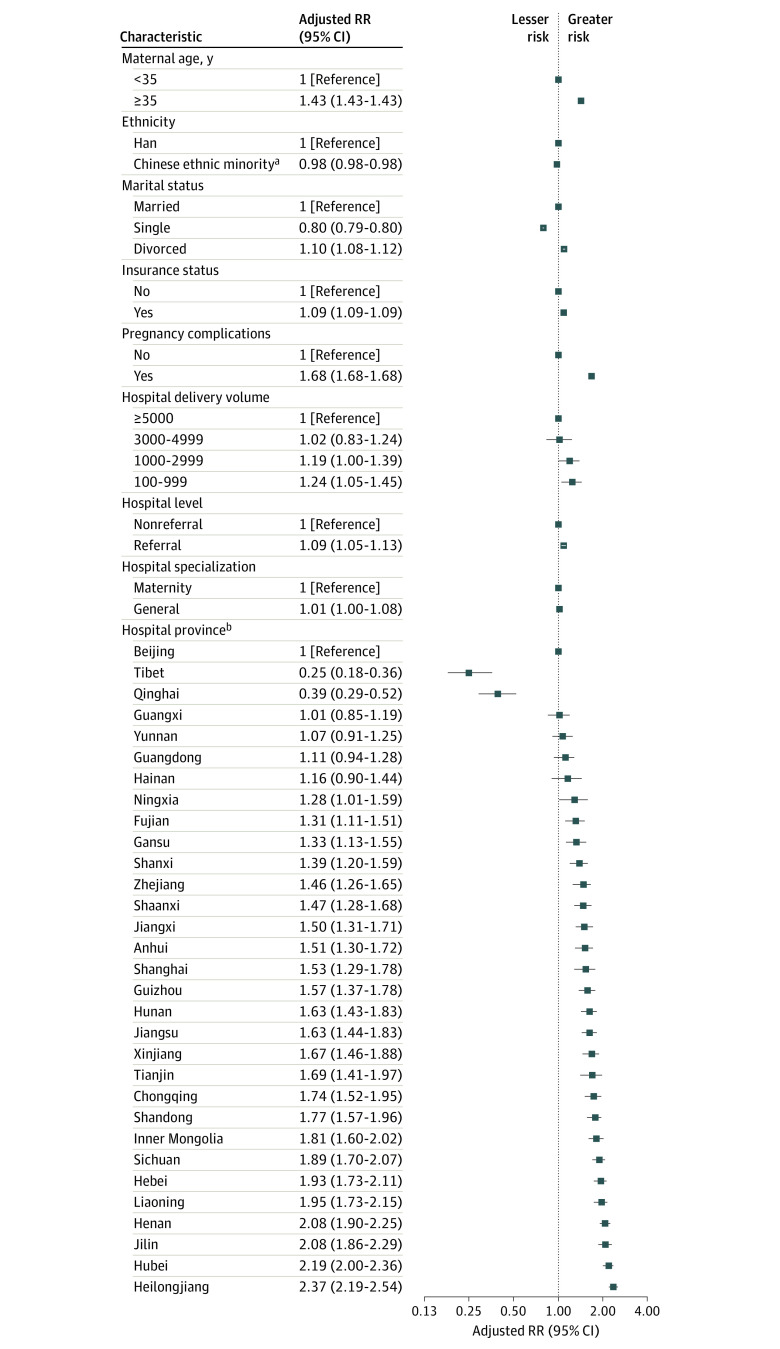
Association of Individual and Hospital Factors With Low-risk Cesarean Delivery Rate in China in 2020 Relative risks (RRs) and corresponding 95% CIs were calculated from hierarchical logistic regression models, with adjustment for maternal age, ethnicity, marital status, medical insurance status, pregnancy complication, hospital level, hospital specialization, hospital delivery volume, and province of hospital location. ^a^Includes Zhuang, Hui, Manchu, Uighur, and other ethnicity. ^b^Four geographic regions were classified based on province of hospital location as follows: northeastern (Heilongjiang, Jilin, and Liaoning), eastern (Beijing, Fujian, Guangdong, Hainan, Hebei, Jiangsu, Shanghai, Tianjin, and Zhejiang), central (Anhui, Henan, Hubei, Hunan, Jiangxi, and Shanxi), and western (Chongqing, Gansu, Guangxi, Guizhou, Inner Mongolia, Ningxia, Qinghai, Shaanxi, Sichuan, Tibet, Xinjiang, and Yunnan).

Among low-risk deliveries, there were several individual and hospital factors associated with risk for cesarean delivery (Figure 2). Of note, hospital province location was associated with risk for cesarean delivery (Figure 2 and eFigure 3 in Supplement 1). For example, compared with hospitals in Beijing, the adjusted risk for cesarean delivery was 137.2% higher in Heilongjiang (adjusted RR, 2.37 [95% CI, 2.19-2.54]), 108.4% higher in Jilin (adjusted RR, 2.08 [95% CI, 1.86-2.29]), and 94.8% higher in Liaoning (adjusted RR, 1.95 [95% CI, 1.73-2.15])—the 3 provinces in the northeastern region of China.

**Figure 3.**
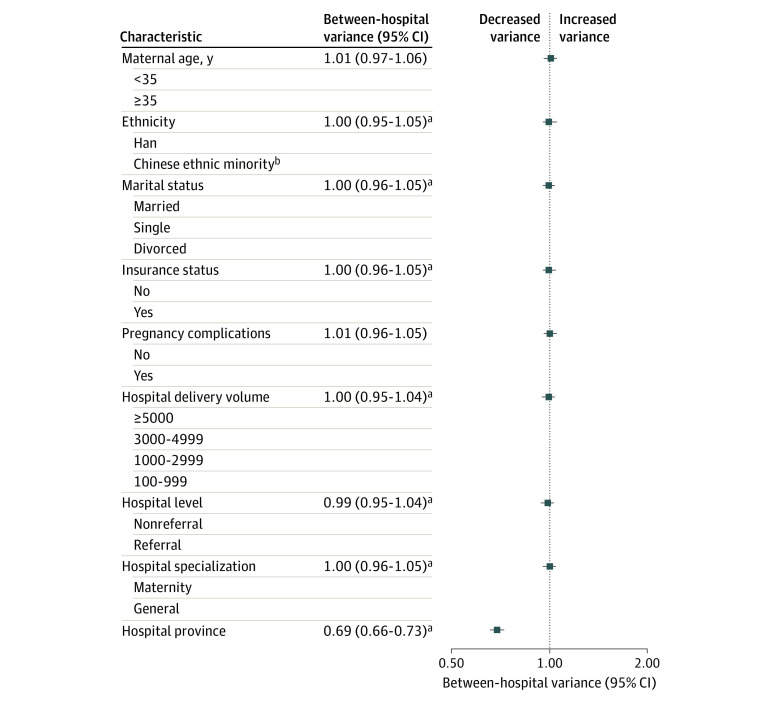
Between-Hospital Variance in Low-risk Cesarean Delivery Rates in China in 2020 ^a^Risk factor results in decreased between-hospital variance. ^b^Includes Zhuang, Hui, Manchu, Uighur, and other ethnicity.

Hospital and individual factors explained 30.8% of hospital variation in cesarean rate, with a reduction in the variance estimate from 1.00 (95% CI, 0.96-1.05) logits in the unadjusted model to 0.69 (95% CI, 0.66-0.73) logits in the fully adjusted model (Hosmer-Lemeshow χ^2^ statistic = 15.61; *P* = .08) (Figure 3). Hospital factors explained 31.3% of hospital variation in the cesarean delivery rate; the largest contribution was the province in which the hospital was located (30.9%), followed by hospital level (1.3%), hospital delivery volume (0.8%), and hospital specialization (0.5%). Individual factors, including ethnicity, marital status, and medical insurance status, additionally explained 2.0% of the variation.

## Discussion

In this cross-sectional study of 7 635 149 deliveries (accounting for 63.0% of all deliveries nationwide) and 6 599 468 low-risk deliveries in 4359 hospitals distributed through 31 provinces in China, the cesarean rate was 44.5% in 2020 and was up to 40.0% even among low-risk deliveries. This study is the first, to our knowledge, to provide data on hospital variation in overall and low-risk cesarean delivery rates in mainland China. Cesarean rates varied markedly among hospitals and were more pronounced for low-risk than overall deliveries. The results of this study suggest that hospital variation in low-risk cesarean rates was more likely to be driven by hospital rather than individual factors.

In this study, the hospital-based overall and low-risk cesarean delivery rates we observed for 2020 (44.5% and 40.0%, respectively) were higher than those for 2016 (41.1% and 35.2%, respectively), which were estimated from 438 hospitals covered by the China National Maternal Near Miss Surveillance System.^5^ These estimates suggest that the cesarean delivery rate in China may have increased after 2016, similar to findings of a population-based study based on the China National Maternal and Child Health Statistics data set.^6^ The low-risk cesarean delivery rate observed in our study was substantially higher than that in the US (25.9% in 2020).^31^ However, our estimate was based on the AHRQ definition, whereas the US estimate was based on the Joint Commission (JC) definition. Both definitions are similar except the AHRQ measure captures nulliparous women and multiparous women with no prior cesarean deliveries, while the JC measure applies only to nulliparous women; therefore, low-risk cesarean rates were comparable between the 2 measures.^15^ However, the low-risk cesarean delivery rate may be greater than 40.0% when using the JC measure, given that one study in China showed a higher cesarean rate in nulliparous than in multiparous women without prior cesarean deliveries.^5^ The cesarean rate in our study was also more than double the threshold targets for improving maternal and child health (15% or 19%),^7,8^ suggesting that cesarean delivery was likely to be overused in China, even among low-risk deliveries. Safely reducing cesarean rates in low-risk deliveries has become a public health focus in many nations. A US study^32^ showed that the cesarean rate for low-risk deliveries decreased in settings in which coordinated hospital-level collaborative and statewide initiatives designed to support vaginal deliveries were implemented. Further research is needed to develop related interventions applicable to the Chinese population.

Between-hospital variation in cesarean rates was large. The absolute difference between the 5th and 95th percentiles was 53.5% for overall deliveries and 56.8% for low-risk deliveries. A previous US study reported an absolute difference of 29% among 1373 hospitals for overall deliveries and 24% for low-risk deliveries during 2009 to 2010.^20^ A UK study reported an absolute difference of 18.3% among 146 National Health Service hospitals in 2008.^19^ Our findings in this study suggest that hospital variation in cesarean rates is greater in China than in the US and UK, reflecting inconsistency in cesarean use among hospitals and indicating both underuse and predominantly overuse of cesarean delivery in China.^17^ It is worth noting that low-risk cesarean rates varied more among hospitals than overall cesarean rates in our study, highlighting greater potential for optimizing cesarean use in low-risk deliveries. Cesarean use in low-risk deliveries was likely affected by some nonobjective factors such as hospital adherence to guidance, clinician attitudes and practices, local culture, and maternal preferences.^17^

In our study, hospital variation in cesarean rates in low-risk deliveries seemed to be driven more by identified hospital factors than individual factors. Hospital factors accounted for 31.3% of the hospital variation in cesarean rate in low-risk deliveries, while individual factors accounted for only 2.0%. Potential causes of variation in cesarean delivery rates are still debatable.^17,19,20^ A UK study indicated that approximately one-third of hospital variation in cesarean rates was attributable to patient characteristics,^19^ while a US study reported that patient diagnoses, sociodemographic factors, and hospital characteristics did not contribute to the variation.^20^ Similar to our findings, another US study showed that hospital location, but not patient characteristics, predominantly contributed to hospital variation.^17^ These differences may have been due to the inclusion of different factors in the studies as well as disparate maternity care management between health care systems in different countries, such as payment structures, out-of-pocket costs, and the role of obstetric care.

The primary contributor to hospital variation in the present study was the province in which the hospital was located, accounting for 30.9%. This finding is similar to a US study that reported hospital geographic location as the primary contributor (39.6%).^17^ Our results are also consistent with previous findings of wide geographic variation in cesarean delivery rates in China,^33^ suggesting that health system factors related to geographic location likely shape hospital variation. For example, different social norms and cultures formulated within a province, different forms and quality of provincial training for obstetricians and midwives, geographic accessibility to facilities with surgical capacity, or financial incentives may affect cesarean delivery rates among hospitals.^20^

### Limitations

This study has some limitations. First, cesarean delivery rates were likely overestimated, as hospitals in rural regions with lower cesarean rates and underdeveloped electronic medical record systems are more likely to underreport data to the HQMS database. In addition, the HQMS did not include primary hospitals that usually have no capacity to perform cesarean procedures. Indeed, our rate was higher than the population-based cesarean delivery rate from the National Maternal and Child Health Statistics data set (36.7% in 2018)^9^ but close to the hospital-based rate from the China National Maternal Near Miss Surveillance System, which disproportionately sampled urban hospitals (41.1% in 2016).^5^

Second, it is possible that inaccuracies in the code biased estimates of cesarean delivery rate and hospital variation in the rate because the definition of cesarean delivery relies on coding accuracy and practices, which may vary among hospitals and provinces. We could not validate the coding of cesarean procedures in the hospital statistics database against hospital records; however, studies in other countries have reported high levels of agreement (κ >0.98).^34,35,36^ Therefore, errors resulting from cesarean coding are unlikely to explain the large hospital variation in cesarean delivery rates observed in our study.

Third, approximately two-thirds of the hospital variation could not be explained, which may have been due to physician factors or additional hospital and individual factors.^37,38^ However, we were unable to examine these potential contributors to the variation, as they were not available in the HQMS database.

Finally, we excluded hospitals with fewer than 100 deliveries in 2020. Therefore, our findings may not generalize to hospitals with small-volume obstetric units.

## Conclusions

In 2020, the cesarean delivery rate in China was 40.0%, even among low-risk deliveries. The rate varied substantially among hospitals and was more pronounced for low-risk than overall deliveries. About one-third of the variation in low-risk deliveries could be explained by hospital rather than individual factors. The current picture of hospital cesarean rates in China suggests a need for hospitals and clinicians to adhere to guidance on cesarean delivery. Our findings may help health administration authorities and policy makers design hospital-level quality improvement interventions to control the overuse of cesarean delivery, particularly for low-risk deliveries. Further research is warranted to determine whether physician factors, hospital policies, or clinical practices contribute to the variation in cesarean delivery rates in China.

## Notes

Supplement 1. **eFigure 1.** Flow Diagram of Pregnant Women in the Hospital Quality Monitoring System (HQMS) in 2020 **eFigure 2.** Funnel Plot of Hospital Cesarean Rates Among Overall Deliveries and Low-Risk Deliveries at 4359 Hospitals in Mainland China in 2020 **eFigure 3.** Cesarean Delivery Rates in Mainland China by Province **eTable 1.** *International Classification of Diseases* (*ICD*) Codes for Cesarean Delivery **eTable 2.** Data From Hierarchical Models for Cesarean Delivery in Low-Risk Deliveries in the Hospital Quality Monitoring System in 2020 **eTable 3. **Distribution of Overall and Low-Risk Cesarean Rates Among Hospitals by Hospital Specialization and Level

Supplement 2.
Data Sharing Statement

## References

[1] Sandall J, Tribe RM, Avery L, . Short-term and long-term effects of caesarean section on the health of women and children. Lancet. 2018;392(10155):1349-1357. doi:10.1016/S0140-6736(18)31930-5 30322585

[2] Keag OE, Norman JE, Stock SJ. Long-term risks and benefits associated with cesarean delivery for mother, baby, and subsequent pregnancies: systematic review and meta-analysis. PLoS Med. 2018;15(1):e1002494. doi:10.1371/journal.pmed.1002494 29360829 PMC5779640

[3] United Nations. World Population Prospects. United Nations Department of Economic and Social Affairs Population Division; 2022.

[4] Lumbiganon P, Laopaiboon M, Gülmezoglu AM, ; World Health Organization Global Survey on Maternal and Perinatal Health Research Group. Method of delivery and pregnancy outcomes in Asia: the WHO Global Survey on Maternal and Perinatal Health 2007-08. Lancet. 2010;375(9713):490-499. doi:10.1016/S0140-6736(09)61870-5 20071021

[5] Liang J, Mu Y, Li X, . Relaxation of the one child policy and trends in caesarean section rates and birth outcomes in China between 2012 and 2016: observational study of nearly seven million health facility births. BMJ. 2018;360:k817. doi:10.1136/bmj.k817 29506980 PMC5836714

[6] Li HT, Hellerstein S, Zhou YB, Liu JM, Blustein J. Trends in cesarean delivery rates in China, 2008–2018. JAMA. 2020;323(1):89-91. doi:10.1001/jama.2019.17595 31910272 PMC6990817

[7] Molina G, Weiser TG, Lipsitz SR, . Relationship between cesarean delivery rate and maternal and neonatal mortality. JAMA. 2015;314(21):2263-2270. doi:10.1001/jama.2015.15553 26624825

[8] World Health Organization. Appropriate technology for birth. Lancet. 1985;2(8452):436-437. 2863457

[9] State Council of the People’s Republic of China. Improving Birth Policies to Promote Long-term and Balanced Population Development. State Council of the People’s Republic of China; 2021.

[10] National Health Commission of China. Notice on Carrying Out the Reassessment of the Baby Friendly Hospitals. National Health Commission of China; 2019.

[11] National Health Commission of China. The Name List of Baby Friendly Hospitals in China. National Health Commission of China; 2015.

[12] National Health Commission of China. Progress Report on Maternal and Child Health in China. National Health Commission of China; 2019.

[13] Zhang Y, Zhou YB, Li HT, . [Secular trends of institutional delivery rate in China from 1996 to 2015]. Zhonghua Yi Xue Za Zhi. 2017;97(17):1337-1342. 28482438 10.3760/cma.j.issn.0376-2491.2017.17.014

[14] Agency for Healthcare Research and Quality. Primary cesarean delivery rate, uncomplicated (IQI 33). HHS:005884. AHRQ National Quality Measures Clearinghouse. Agency for Healthcare Research and Quality; 2015.

[15] Armstrong JC, Kozhimannil KB, McDermott P, Saade GR, Srinivas SK; Society for Maternal-Fetal Medicine Health Policy Committee. Comparing variation in hospital rates of cesarean delivery among low-risk women using 3 different measures. Am J Obstet Gynecol. 2016;214(2):153-163. doi:10.1016/j.ajog.2015.10.935 26593970

[16] Muza S. The current state of birth in Australia. 2020. Accessed November 24, 2020. https://www.lamaze.org/Giving-Birth-with-Confidence/GBWC-Post/the-current-state-of-birth-in-australia

[17] Sebastião YV, Womack L, Vamos CA, . Hospital variation in cesarean delivery rates: contribution of individual and hospital factors in Florida. Am J Obstet Gynecol. 2016;214(1):123.e1-123.e18. doi:10.1016/j.ajog.2015.08.027 26292046

[18] Pasko DN, McGee P, Grobman WA, ; Eunice Kennedy Shriver National Institute of Child Health and Human Development (NICHD) Maternal-Fetal Medicine Units (MFMU) Network. Variation in the nulliparous, term, singleton, vertex cesarean delivery rate. Obstet Gynecol. 2018;131(6):1039-1048. doi:10.1097/AOG.0000000000002636 29742665 PMC6033063

[19] Bragg F, Cromwell DA, Edozien LC, . Variation in rates of caesarean section among English NHS trusts after accounting for maternal and clinical risk: cross sectional study. BMJ. 2010;341:c5065. doi:10.1136/bmj.c5065 20926490 PMC2950923

[20] Kozhimannil KB, Arcaya MC, Subramanian SV. Maternal clinical diagnoses and hospital variation in the risk of cesarean delivery: analyses of a National US Hospital Discharge Database. PLoS Med. 2014;11(10):e1001745. doi:10.1371/journal.pmed.1001745 25333943 PMC4205118

[21] Jiang L, Krumholz HM, Li X, Li J, Hu S. Achieving best outcomes for patients with cardiovascular disease in China by enhancing the quality of medical care and establishing a learning health-care system. Lancet. 2015;386(10002):1493-1505. doi:10.1016/S0140-6736(15)00343-8 26466053 PMC5323019

[22] Han X, Zhou H. Monitoring traumatic brain injury in China. Lancet Neurol. 2019;18(9):813. doi:10.1016/S1474-4422(19)30237-6 31397282

[23] Wang Y, Shi H, Chen L, . Absolute risk of adverse obstetric outcomes among twin pregnancies after in vitro fertilization by maternal age. JAMA Netw Open. 2021;4(9):e2123634. doi:10.1001/jamanetworkopen.2021.23634 34505887 PMC8433605

[24] Zhang L, Zhao MH, Zuo L, ; CK-NET Work Group. China Kidney Disease Network (CK-NET) 2015 Annual Data Report. Kidney Int Suppl (2011). 2019;9(1):e1-e81. doi:10.1016/j.kisu.2018.11.001 30828481 PMC6382959

[25] Wang L, Xu X, Baker P, . Patterns and associated factors of caesarean delivery intention among expectant mothers in China: implications from the implementation of China’s new national two-child policy. Int J Environ Res Public Health. 2016;13(7):686. doi:10.3390/ijerph13070686 27399752 PMC4962227

[26] National Health Commission. China Health Statistics Yearbook 2021. Peking Union Medical College Press; 2021.

[27] Xiao CM, Sun JW, Ye ZY. Evolution of China’s regional economic development strategy. Study and Practice. 2010;(7):5-11.

[28] Main EK, Chang SC, Cheng YW, Rosenstein MG, Lagrew DC. Hospital-level variation in the frequency of cesarean delivery among nulliparous women who undergo labor induction. Obstet Gynecol. 2020;136(6):1179-1189. doi:10.1097/AOG.0000000000004139 33156193

[29] Spiegelhalter DJ. Funnel plots for comparing institutional performance. Stat Med. 2005;24(8):1185-1202. doi:10.1002/sim.1970 15568194

[30] Zhang J, Yu KF. What’s the relative risk? A method of correcting the odds ratio in cohort studies of common outcomes. JAMA. 1998;280(19):1690-1691. doi:10.1001/jama.280.19.1690 9832001

[31] US Department of Health and Human Services, Office of Disease Prevention and Health Promotion. Reduce cesarean births among low-risk women with no prior births—MICH-06. 2020. Accessed May 25, 2022. https://health.gov/healthypeople/objectives-and-data/browse-objectives/pregnancy-and-childbirth/reduce-cesarean-births-among-low-risk-women-no-prior-births-mich-06

[32] Rosenstein MG, Chang SC, Sakowski C, . Hospital quality improvement interventions, statewide policy initiatives, and rates of cesarean delivery for nulliparous, term, singleton, vertex births in California. JAMA. 2021;325(16):1631-1639. doi:10.1001/jama.2021.3816 33904868 PMC8080226

[33] Li HT, Luo S, Trasande L, . Geographic variations and temporal trends in cesarean delivery rates in China, 2008–2014. JAMA. 2017;317(1):69-76. doi:10.1001/jama.2016.18663 28030701

[34] Yasmeen S, Romano PS, Schembri ME, Keyzer JM, Gilbert WM. Accuracy of obstetric diagnoses and procedures in hospital discharge data. Am J Obstet Gynecol. 2006;194(4):992-1001. doi:10.1016/j.ajog.2005.08.058 16580288

[35] Tajima K, Ishikawa T, Matsuzaki F, . Validity of administrative data for identifying birth-related outcomes with the end date of pregnancy in a Japanese university hospital. Int J Environ Res Public Health. 2022;19(8):4864. doi:10.3390/ijerph19084864 35457731 PMC9025717

[36] Josberger RE, Wu M, Nichols EL. Birth certificate validity and the impact on primary cesarean section quality measure in New York State. J Community Health. 2019;44(2):222-229. doi:10.1007/s10900-018-0577-y 30324538

[37] Reime B, Klein MC, Kelly A, . Do maternity care provider groups have different attitudes towards birth? BJOG. 2004;111(12):1388-1393. doi:10.1111/j.1471-0528.2004.00338.x 15663124

[38] Luo ZC, Liu X, Wang A, . Obstetricians’ perspectives on trial of labor after cesarean (TOLAC) under the two-child policy in China: a cross-sectional study. BMC Pregnancy Childbirth. 2021;21(1):89. doi:10.1186/s12884-021-03559-1 33509100 PMC7841882

