# Supplementary material for: Evaluation of Cesarean Rates for Term, Singleton, Live Vertex Deliveries in China in 2020 Among Women With No Prior Cesarean Delivery
Source: JAMA Netw Open. 2023 Mar 23;6(3):e234521. doi: 10.1001/jamanetworkopen.2023.4521 (PMC10037159; doi:10.1001/jamanetworkopen.2023.4521)
Supplement: Supplement 1. — eFigure 1. Flow Diagram of Pregnant Women in the Hospital Quality Monitoring System (HQMS) in 2020 eFigure 2. Funnel Plot of Hospital Cesarean Rates Among Overall Deliveries and Low-Risk Deliveries at 4359 Hospitals in Mainland China in 2020 eFigure 3. Cesarean Delivery Rates in Mainland China by Province eTable 1. International Classification of Diseases (ICD) Codes for Cesarean Delivery eTable 2. Data From Hierarchical Models for Cesarean Delivery in Low-Risk Deliveries in the Hospital Quality Monitoring System in 2020 eTable 3. Distribution of Overall and Low-Risk Cesarean Rates Among Hospitals by Hospital Specialization and Level [file jamanetwopen-e234521-s001.pdf]

Supplementary Online Content

Yin S, Chen L, Zhou Y, et al. Evaluation of cesarean rates for term, singleton, live vertex deliveries in China in 2020 among women with no prior cesarean delivery. *JAMA Netw Open*. 2023;6(3):e234521. doi:10.1001/jamanetworkopen.2023.4521

eFigure 1. Flow Diagram of Pregnant Women in the Hospital Quality Monitoring System (HQMS) in 2020

eFigure 2. Funnel Plot of Hospital Cesarean Rates Among Overall Deliveries and Low-Risk Deliveries at 4359 Hospitals in Mainland China in 2020

eFigure 3. Cesarean Delivery Rates in Mainland China by Province

eTable 1. *International Classification of Diseases* (*ICD*) Codes for Cesarean Delivery

eTable 2. Data From Hierarchical Models for Cesarean Delivery in Low-Risk Deliveries in the Hospital Quality Monitoring System in 2020

eTable 3. Distribution of Overall and Low-Risk Cesarean Rates Among Hospitals by Hospital Specialization and Level

This supplementary material has been provided by the authors to give readers additional information about their work.

© 2023 Yin S et al. *JAMA Network Open*.

eFigure 1. Flow Diagram of Pregnant Women in the Hospital Quality Monitoring System (HQMS) in 2020

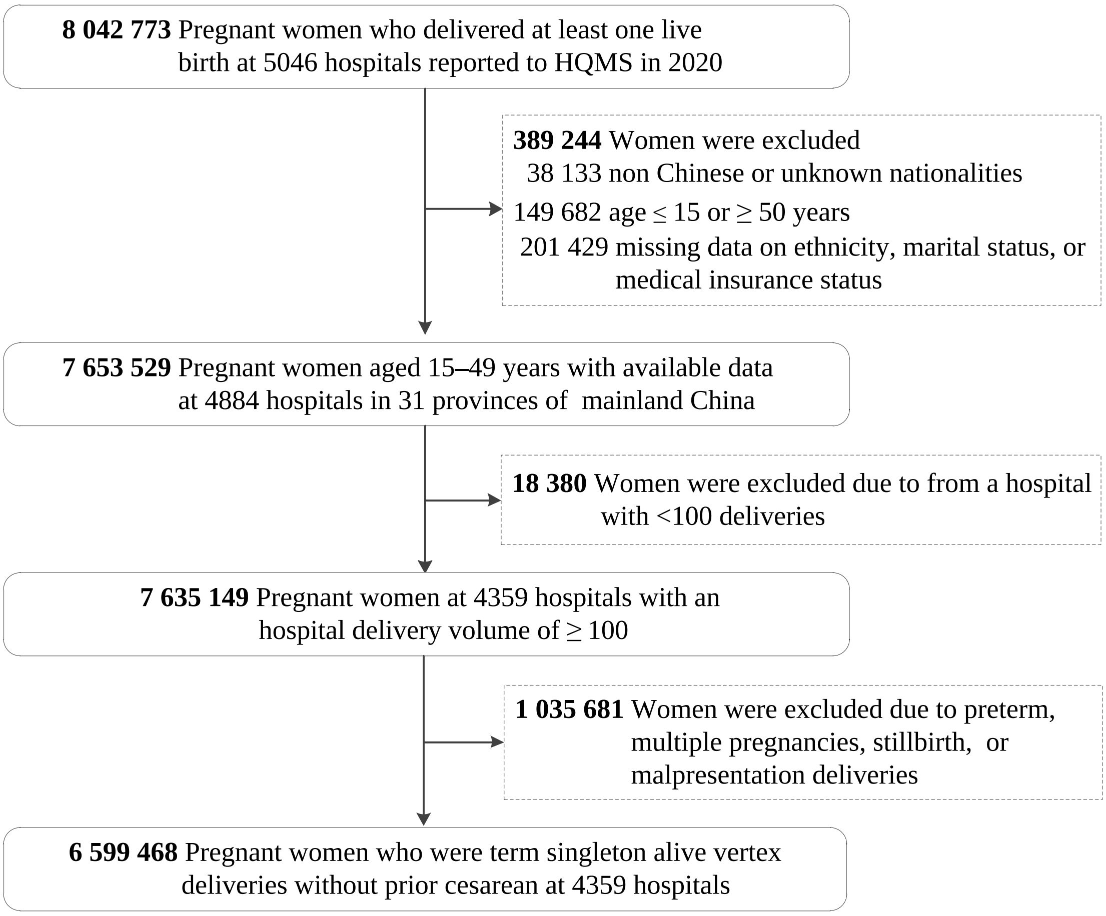

© 2023 Yin S et al. *JAMA Network Open*.

eFigure 2. Funnel Plot of Hospital Cesarean Rates Among Overall Deliveries and Low-Risk Deliveries at 4359 Hospitals in Mainland China in 2020

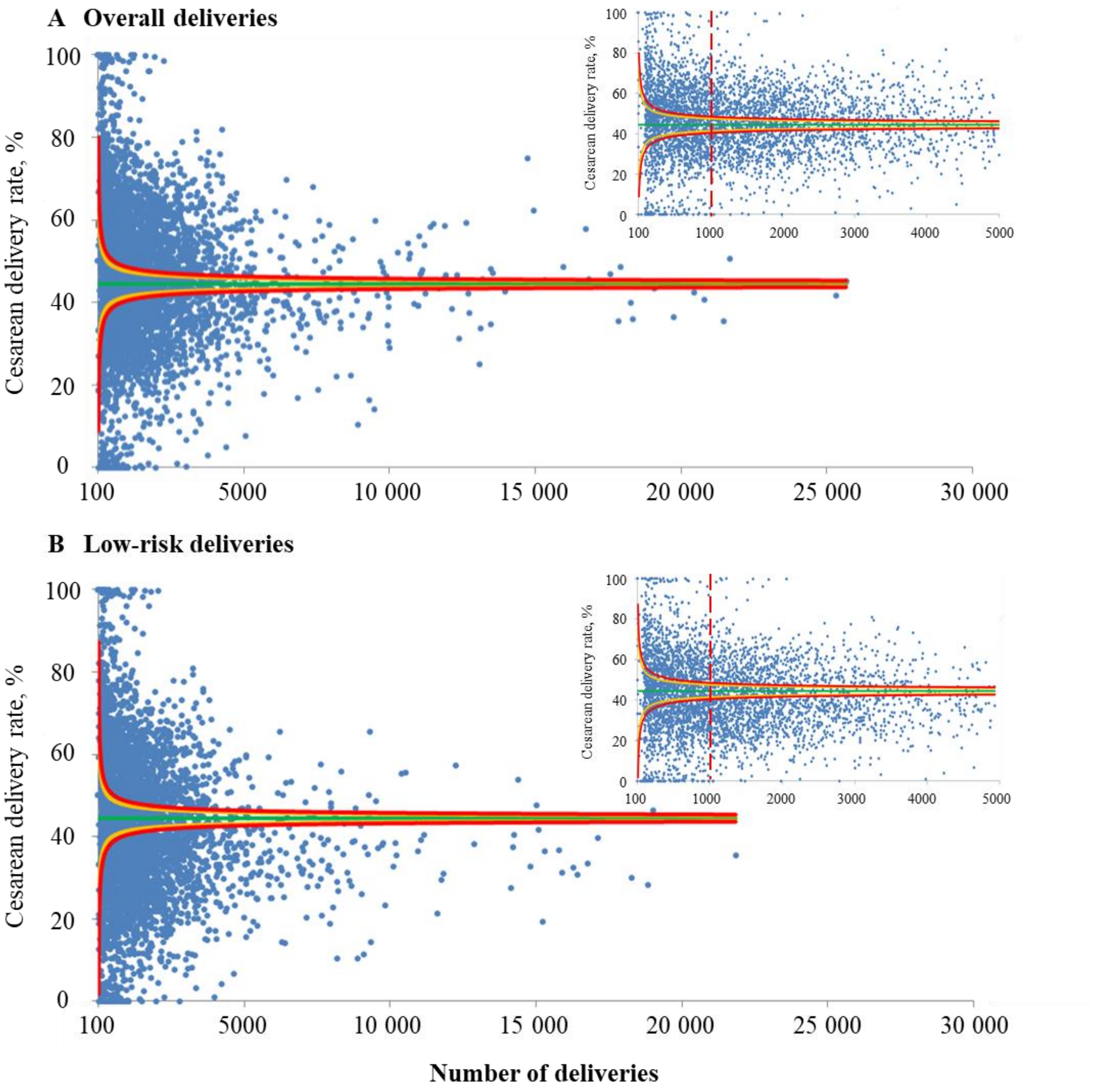

Funnel plots show how each hospital (blue dot) performs compared to the mean (green) and the 95% (yellow) and 99% control limits (red). Inserts in the upper right of each sub-figure represent zoom-in on the x-axis to allow visualization of the cesarean rates corresponding to hospitals with 100–50000 deliveries.

© 2023 Yin S et al. *JAMA Network Open*.

eFigure 3. Cesarean Delivery Rates in Mainland China by Province

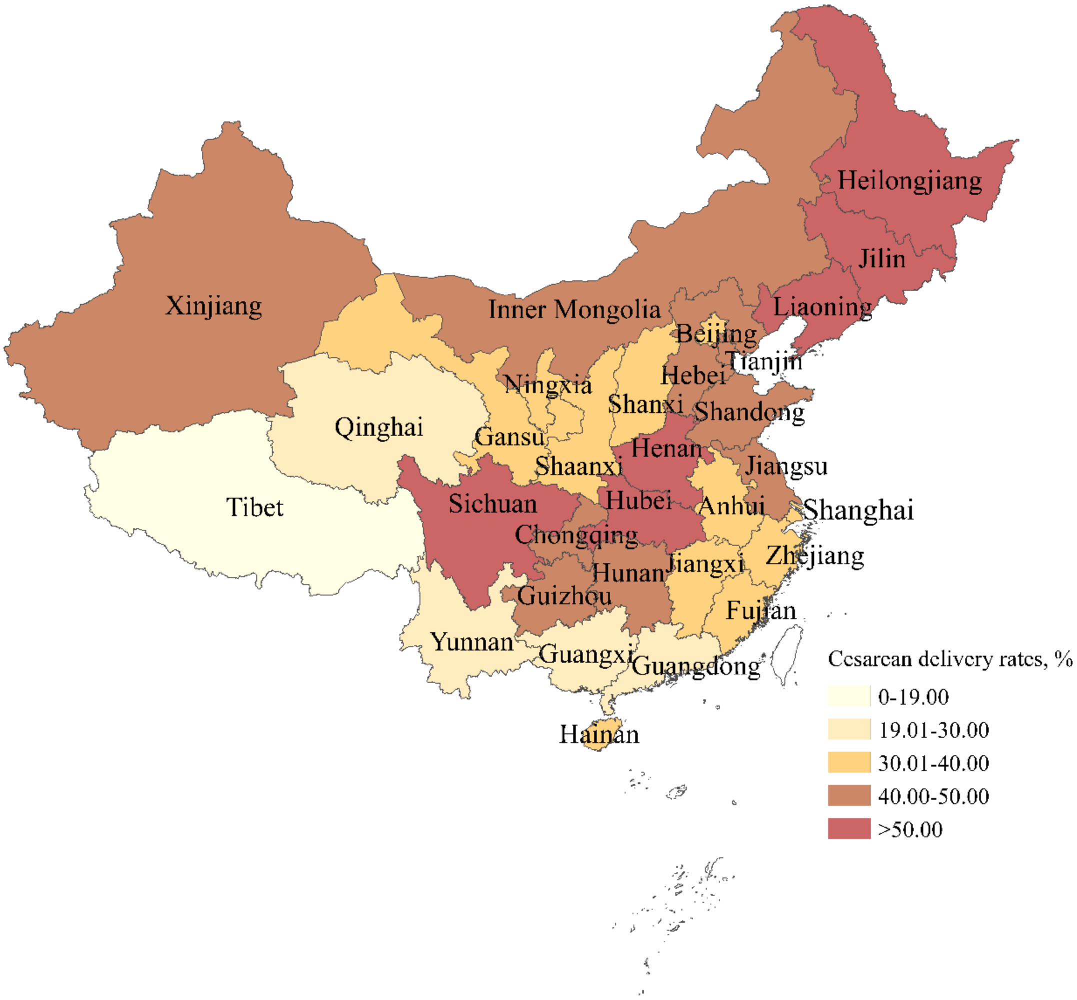

© 2023 Yin S et al. *JAMA Network Open*.

eTable 1. *International Classification of Diseases* (*ICD*) Codes for Cesarean Delivery

ICD-sets	ICD-code/ Diagnosis or Operations codes
**National standard version 1.0-ICD10**	
	O82.100, O82.200, O82.800, O82.900, O84.200, O86.002, O90.000, P03.400, P03.401
**National clinical version 1.0-ICD10**	
	O60.100x002, O60.300x001, O82.800, O82.900, O82.900x001, O84.200, O86.002, O86.000x002, O86.000x003, O86.800x001, O90.000, O90.000x001, O90.000x002, O90.000x003, P03.400, P03.401
**Beijing version 4.0-ICD10**	
	O60.102, O60.201, O60.301, O82.001, O82.101, O82.201, O82.801, O82.901, O84.201, O86.801, O86.002, O90.001, O90.002
**Beijing version of procedures/operations ICD-9-CM**	
	74.0x00, 74.1x00, 74.1x01, 74.1x02, 74.2x00, 74.4x00, 74.9900
**National clinical version of procedures/operations 1.1- ICD-9-CM**	
	74.0 001, 74.1 001, 74.1 002, 74.1 003, 74.2 002

© 2023 Yin S et al. *JAMA Network Open*.

eTable 2. Data From Hierarchical Models for Cesarean Delivery in Low-Risk Deliveries in the Hospital Quality Monitoring System in 2020

	Cesarean rate (%)	Odds ratio (95% confidence interval)				Relative risk (95% confidence interval)	
		Crude	*P*-crude	Adjusted	*P*-adjusted	Crude	Adjusted
**Maternal age, y**							
<35	38.0	1 [Reference]		1 [Reference]		1 [Reference]	1 [Reference]
≥35	55.6	2.12 (2.11-2.13)	<0.001	1.94 (1.93-1.95)	<0.001	1.49 (1.48-1.49)	1.43 (1.43-1.43)
**Ethnicity**							
Han	40.6	1 [Reference]		1 [Reference]		1 [Reference]	1 [Reference]
Ethnic minorities	34.4	0.96 (0.96-0.97)	<0.001	0.97 (0.96-0.97)	<0.001	0.98 (0.98-0.98)	0.98 (0.98-0.98)
**Marital status**							
Married	40.4	1 [Reference]		1 [Reference]		1 [Reference]	1 [Reference]
Single	28.9	0.64 (0.64-0.65)	<0.001	0.70 (0.69-0.70)	<0.001	0.75 (0.75-0.76)	0.80 (0.79-0.80)
Divorced	46.4	1.35 (1.31-1.39)	<0.001	1.18 (1.14-1.22)	<0.001	1.18 (1.16-1.20)	1.10 (1.08-1.12)
**Medical insurance status**							
No	38.1	1 [Reference]		1 [Reference]		1 [Reference]	1 [Reference]
Yes	40.9	0.87 (0.86-0.87)	<0.001	1.15 (1.15-1.16)	<0.001	0.92 (0.91-0.92)	1.09 (1.09-1.09)
**Pregnancy complications** [a]							
No	35.1	1 [Reference]		1 [Reference]		1 [Reference]	1 [Reference]
Yes	58.9	2.75 (2.74-2.76)	<0.001	2.65 (2.64-2.66)	<0.001	1.70 (1.70-1.71)	1.68 (1.68-1.68)
**Hospital delivery volume, deliveries**							
≥5000	36.9	1 [Reference]		1 [Reference]		1 [Reference]	1 [Reference]
3000–4999	35.9	1.04 (0.80,1.35)	0.76	1.02 (0.86-1.20)	0.58	0.95 (0.73-1.20)	1.02 (0.83-1.24)
1000–2999	40.4	1.18 (0.91-1.53)	0.21	1.11 (0.94-1.28)	0.06	1.08 (0.86-1.31)	1.19 (1.00-1.39)
100–999	42.9	1.30 (1.00-1.69)	0.05	1.17 (1.00-1.35)	0.02	1.45 (0.91-1.37)	1.24 (1.05-1.45)

© 2023 Yin S et al. *JAMA Network Open*.

**Hospital level**							
Non-referral	38.2	1 [Reference]		1 [Reference]		1 [Reference]	1 [Reference]
Referral	41.4	1.27 (1.19-1.35)	<0.001	1.15 (1.08-1.21)	<0.001	1.15 (1.11-1.19)	1.09 (1.05-1.13)
**Hospital specialization**							
Maternity-specialized	37.7	1 [Reference]		1 [Reference]		1 [Reference]	1 [Reference]
General	40.8	1.10 (1.01-1.20)	0.03	1.05 (1.00-1.13)	0.02	1.06 (1.01-1.12)	1.01 (1.00-1.08)
**Hospital location[b]**							
Beijing	30.4	1 [Reference]		1 [Reference]		1 [Reference]	1 [Reference]
Anhui	38.5	1.46 (1.13-1.89)	<0.001	1.94 (1.50-2.52)	<0.001	1.28 (1.09-1.49)	1.51 (1.30-1.72)
Chongqing	44.7	1.88 (1.44-2.45)	<0.001	2.56 (1.96-3.35)	<0.001	1.48 (1.27-1.70)	1.74 (1.52-1.95)
Fujian	32.5	1.06 (0.82-1.38)	0.64	1.51 (1.16-1.95)	<0.001	1.04 (0.87-1.24)	1.31 (1.11-1.51)
Gansu	33.2	1.10 (0.84-1.43)	0.49	1.56 (1.19-2.03)	<0.001	1.07 (0.88-1.26)	1.33 (1.13-1.55)
Guangdong	29.4	0.81 (0.64-1.01)	0.06	1.16 (0.92-1.45)	0.29	0.86 (0.72-1.01)	1.11 (0.94-1.28)
Guangxi	26.6	0.72 (0.57-0.92)	<0.001	1.01 (0.80-1.29)	0.88	0.79 (0.66-0.94)	1.01 (0.85-1.19)
Guizhou	40.1	1.50 (1.17-1.93)	<0.001	2.10 (1.63-2.70)	<0.001	1.30 (1.11-1.50)	1.57 (1.37-1.78)
Hainan	30.8	0.89 (0.62-1.28)	0.54	1.24 (0.86-1.79)		0.92 (0.70-1.18)	1.16 (0.90-1.44)
Hebei	47.0	2.39 (1.89-3.02)	<0.001	3.23 (2.55-4.09)	<0.001	1.68 (1.49-1.87)	1.93 (1.73-2.11)
Henan	50.8	2.73 (2.17-3.44)	<0.001	3.95 (3.14-4.98)	<0.001	1.79 (1.60-1.98)	2.08 (1.90-2.25)
Heilongjiang	59.0	4.72 (3.61-6.17)	<0.001	5.93 (4.53-7.76)	<0.001	2.22 (2.01-2.40)	2.37 (2.19-2.54)
Hubei	58.2	3.53 (2.76-4.52)	<0.001	4.56 (3.56-5.84)	<0.001	2.00 (1.80-2.18)	2.19 (2.00-2.36)
Hunan	40.9	1.71 (1.34-2.19)	<0.001	2.25 (1.76-2.88)	<0.001	1.41 (1.21-1.61)	1.63 (1.43-1.83)
Jilin	54.5	3.01 (2.26-4.00)	<0.001	3.95 (2.97-5.27)	<0.001	1.87 (1.63-2.09)	2.08 (1.86-2.29)
Jiangsu	42.7	1.77 (1.39-2.26)	<0.001	2.26 (1.78-2.88)	<0.001	1.43 (1.24-1.63)	1.63 (1.44-1.83)
Jiangxi	36.2	1.36 (1.06-1.74)	<0.001	1.93 (1.51-2.48)	<0.001	1.23 (1.04-1.42)	1.50 (1.31-1.71)
Liaoning	50.9	2.69 (2.06-3.51)	<0.001	3.33 (2.55-4.34)	<0.001	1.78 (1.56-1.99)	1.95 (1.73-2.15)
Inner Mongolia	46.3	2.19 (1.69-2.85)	<0.001	2.81 (2.16-3.65)	<0.001	1.61 (1.40-1.82)	1.81 (1.60-2.02)

© 2023 Yin S et al. *JAMA Network Open*.

Ningxia	36.4	1.11 (0.76-1.61)	0.60	1.46 (1.01-2.13)	<0.001	1.07 (0.82-1.36)	1.28 (1.01-1.59)
Qinghai	23.4	0.23 (0.17-0.32)	<0.001	0.31 (0.22-0.43)	<0.001	0.30 (0.23-0.40)	0.39 (0.29-0.52)
Shandong	44.7	2.04 (1.62-2.59)	<0.001	2.66 (2.10-3.37)	<0.001	1.55 (1.36-1.75)	1.77 (1.57-1.96)
Shanxi	36.5	1.25 (0.98-1.59)	0.08	1.68 (1.31-2.15)	<0.001	1.16 (0.99-1.35)	1.39 (1.20-1.59)
Shaanxi	40.0	1.39 (1.09-1.77)	<0.001	1.86 (1.45-2.38)	<0.001	1.24 (1.06-1.43)	1.47 (1.28-1.68)
Shanghai	36.1	1.64 (1.22-2.21)	<0.001	1.99 (1.48-2.69)	<0.001	1.37 (1.14-1.62)	1.53 (1.29-1.78)
Sichuan	50.9	2.44 (1.93-3.07)	<0.001	3.08 (2.45-3.88)	<0.001	1.70 (1.50-1.88)	1.89 (1.70-2.07)
Tianjin	47.1	2.10 (1.50-2.96)	<0.001	2.43 (1.72-3.41)	<0.001	1.57 (1.30-1.85)	1.69 (1.41-1.97)
Tibet	15.3	0.14 (0.09-0.20)	<0.001	0.19 (0.13-0.28)	<0.001	0.19 (0.12-0.26)	0.25 (0.18-0.36)
Xinjiang	44.2	1.81 (1.40-2.33)	<0.001	2.35 (1.82-3.04)	<0.001	1.45 (1.25-1.66)	1.67 (1.46-1.88)
Yunnan	28.5	0.81 (0.64-1.03)	0.08	1.10 (0.87-1.40)	0.25	0.86 (0.72-1.02)	1.07 (0.91-1.25)
Zhejiang	36.3	1.39 (1.09-1.77)	<0.001	1.82 (1.43-2.31)	<0.001	1.24 (1.06-1.43)	1.46 (1.26-1.65)

[a] Pregnancy complications included gestational hypertension, pre-eclampsia, placental accreta, placenta previa, placental abruption, antenatal hemorrhage, birth canal morphologic deformity, prolapse of cord, macrosomia, and others.

[b] Four geographic regions were classified based on province of hospital location, as northeastern region included Heilongjiang, Jilin, and Liaoning provinces, eastern region included Beijing, Fujian, Guangdong, Hainan, Hebei, Jiangsu, Shanghai, Tianjin, and Zhejiang provinces, central region included Anhui, Henan, Hubei, Hunan, Jiangxi, and Shanxi provinces, and western region included Chongqing, Gansu, Guangxi, Guizhou, Inner Mongolia, Ningxia, Qinghai, Shaanxi, Sichuan, Tibet, Xinjiang, and Yunnan provinces.

© 2023 Yin S et al. *JAMA Network Open*.

eTable 3. Distribution of Overall and Low-Risk Cesarean Rates Among Hospitals by Hospital Specialization and Level

	Percentiles (%)					
	P5	P25	P50	P75	P95	Difference[a]
**Overall**						
**Maternity-specialized**						
Non-referral	6.2	32.5	41.8	53.5	74.0	67.8
Referral	28.5	38.4	45.0	54.4	68.5	40.0
**General**						
Non-referral	14.6	32.2	43.1	54.0	72.0	57.4
Referral	30.3	41.7	49.8	59.1	73.1	42.8
**Low-risk deliveries**						
**Maternity-specialized**						
Non-referral	5.6	26.6	37.3	51.2	73.6	68.0
Referral	20.3	31.4	38.5	51.1	64.9	44.6
**General**						
Non-referral	11.1	27.9	39.7	51.8	71.7	60.6
Referral	23.2	35.6	45.1	55.2	70.4	47.2

[a]Difference was calculated by subtracting the 5th percentile from the 95th percentile.

© 2023 Yin S et al. *JAMA Network Open*.
