# Supplementary material for: Evaluation of Cesarean Rates for Term, Singleton, Live Vertex Deliveries in China in 2020 Among Women With No Prior Cesarean Delivery
Source: JAMA Netw Open. 2023 Mar 23;6(3):e234521. doi: 10.1001/jamanetworkopen.2023.4521 (PMC10037159; doi:10.1001/jamanetworkopen.2023.4521)
Supplement: Supplement 2. — Data Sharing Statement [file jamanetwopen-e234521-s002.pdf]

Data Sharing Statement

Yin. Evaluation of Cesarean Rates for Term, Singleton, Live Vertex Deliveries in China in 2020 Among Women With No Prior Cesarean Delivery. *JAMA Netw Open*. Published March 23, 2023. doi:10.1001/jamanetworkopen.2023.4521

Data
Data available: No

Additional Information
Explanation for why data not available: Individual data in this study are not publicly available for ethical and legal reasons. Requests for data should be directed to the corresponding author. Some data can be made available upon reasonable request.
